# Group Homes and COVID-19: Perspectives of Youth Residents, Staff, and Caregivers

**DOI:** 10.3390/ijerph19158978

**Published:** 2022-07-23

**Authors:** Whitney Howey, Andrea Assadollahi, Brad Lundahl

**Affiliations:** College of Social Work, University of Utah, Salt Lake City, UT 84112, USA; andreaassadollahi@gmail.com (A.A.); brad.lundahl@utah.edu (B.L.)

**Keywords:** group home, COVID-19, youth residents, staff, caregivers

## Abstract

Objective: This study explored the perspectives of being in a youth group home during the COVID-19 pandemic from youth residents, staff, and caregivers. Methods: We conducted semi-structured interviews with 9 youth residents, 8 group home staff members, and 13 caregivers of residents. All participants were connected to the group home before and during the COVID-19 pandemic. Thematic analysis was used to identify lived experience themes. Results: Two overarching themes were identified among the youth residents—*Safety response to COVID-19* and *Socialization changes due to COVID*—along with three subthemes: *Structure leading to separation, Support and belonging amid a pandemic,* and *Competency.* Three overarching themes were identified among the group home staff: *Safety response to COVID-19, Increased responsibility,* and *Mental health changes because of a pandemic.* Finally, three overarching themes were identified among the guardians of youth residents: *Safety response to COVID-19, Belief in a mental health impact on the child,* and *Communication during a pandemic.* Conclusions: The findings provide the experiences among three group home stakeholders. Overall, they demonstrated resilience in a setting and time when resilience was essential. Finally, the findings offer insight on the basis of which group homes/organizations can prepare for crises of a great magnitude, including vital communication elements.

## 1. Introduction

COVID-19 has infected and impacted millions of people around the world. The early data suggest that the pandemic had devastating effects on our communities, including youth [1]. Over 12.9 million children and youth tested positive for the virus since the beginning of the pandemic [2] Youth in congregate care settings are at an increased risk of contracting and spreading COVID-19 to others [3], as well as experiencing negative effects because of the virus [1].

Like much of the world, group homes went on lockdown during the early days of COVID-19, meaning residents were confined to group homes and denied outside social interactions, including with family, without clear notions of when visits would be reestablished or if treatment would be prematurely terminated. Group home staff were asked to work despite relatively unknown risk factors in environments where many common activities were restricted, such as therapy groups and recreational activities.

In this qualitative study, we sought to understand the lived experiences of stakeholders in a tertiary residential center treating youth who had sexually assaulted other youth. Our study sought those who experienced the group home both prior to COVID-19 and during the pandemic. This study is informed by elements of the Attachment, Self-regulation, and Competency Model (ARC) and social constructivism. The ARC model provides an intervention framework for children coping with difficult experiences that have interfered with healthy development [4]. Within this framework, there are three levels of interventions: the child, the child’s family or caregivers, and the system culture [4]. The ARC model highlights individualized programs for children to support their needs and the importance of system-wide changes to support effective outcomes [5]. In combination with the ARC model, social constructivism provides a guiding framework for this study. Social constructivism highlights lived experiences and how lived experiences are constructed based on social interactions.

To date, only a few studies have explored the impacts and experiences in residential care for youth with mental health problems during the COVID-19 pandemic. However, stakeholders in residential care are likely to be impacted by the pandemic. For example, Wilke et al. [6] explored the government-mandated rapid return of youth from a residential care setting to their families in response to COVID-19. They found that youth and families were not prepared for the rapid transition, and only some of the families would be able to remain safely intact due to unresolved concerns and other factors that initially placed them in residential care [6].

Parry and colleagues [7] explored the experiences of workers in a youth residential setting and found that providing additional supportive measures was essential for the staff’s well-being. Few studies have included youth perspectives in residential settings during the pandemic. Children in one study expressed concerns for the safety and well-being of their family members, as well as frustration with not having contact with their family [8]. Other children reported better relationships with caregivers and that having a person of trust was relevant to their well-being during the pandemic [9]. Finally, a study conducted by Costa and colleagues [10] found that higher levels of perceived cohesion among youth in residential institutions related to emotional distress stability over time during COVID-19. Overall, the literature has captured the adaptations made by stakeholders and the changes for youth and staff in residential settings in response to the pandemic.

The first and second authors (W.H. & A.A.) were working at the group home before and during the pandemic. We witnessed first-hand the responses from youth, staff, and guardians. We recognize that our experiences as clinicians in the group home during a global pandemic were vastly different from the other roles we sought to better understand. As a doctoral student, one author (W.H.) wondered what research might inform best practices given the unique COVID-19 impact. No related research was identified. This study offers an initial exploration into how youth, their guardians/parents, and staff experienced a residential group home during a global pandemic. From this study, we hope to share their voices, construct knowledge and meaning, and understand the different facets of their lived experiences.

## 2. Method

A qualitative approach was used to explore and better understand the experiences of group home residents, their guardians, and staff during COVID-19. We used semi-structured interviews to collect information. Institutional review board approval was obtained from the University of Utah prior to recruitment, sampling, and interview procedures.

### 2.1. Participants

The eligible participants were residents, residents’ parents or guardians, and staff in a group home serving male youth between the ages of 16 and 20 years. Because we were curious about how stakeholders experienced changes resulting from the pandemic, the participants needed to be involved with the group home before the pandemic (before March 2020) and during the pandemic (after March 2020). Convenience sampling was used to identify participants, which included 9 youth residents, 8 staff member, and 13 parents or guardians. The guardians and youth did not need to be related to be included.

Admission records, discharge records, and employment records were reviewed to identify qualifying participants. The adult qualifying participants and guardians of qualifying youth participants were contacted in person or via an email explaining the study and notifying them of a follow-up call in 7 days regarding the study. Informed consent, including verbatim mandatory reporting language and requirements, was obtained from the participants. Once the guardians provided informed consent for their child, the youth participants were provided with an assent form and were given the opportunity to ask questions or refuse participation. Compensation was not offered.

The principal investigator, pursuing a Ph.D. in social work and having extensive clinical experience working with adolescents, conducted in-depth interviews between March and June 2021; thus, some participants had already been discharged. Interviews were audio-recorded and lasted approximately 60 min. Confidentiality was ensured by conducting interviews in a private setting. Prior to the interview, the purpose of the interview was explained, including the option to withdraw at any time.

The interview questions were designed to gain insight into the perspectives, experiences, and impacts of the pandemic in a group home setting. The guiding questions included: “What is it like living in a group home during a pandemic?”; “What is it like working in a group home during a pandemic?”; “What has it been like having a child in a group home during a pandemic?”; “How has the group home been different since the pandemic began?”; “Have the services been different?” “If so, how?”; “Has work been different?”; “If so, how?”; and “What differences have you directly observed or been told about as a guardian of a resident in the group home?”. Once a question was asked, the interviewer would use minimal prompts to encourage the participant to continue speaking until they seemed done with a given question stem.

### 2.2. Data Analysis

The responses were transcribed verbatim for thematic analyses. Thematic analysis [11] was utilized to identify themes related to the group home and changes due to the pandemic. First, two authors (W.H. and A.A.) read the transcriptions to familiarize themselves with the data. Using ling-by-line coding, initial codes were generated for the 30 transcripts. The codes were then grouped as potential themes within participant groups and across all participants. Finally, themes and subthemes were defined, named, and supported with direct quotes. To ensure accuracy and rigor in the analysis, any disagreements between coders were resolved by further discussion, involving a third reviewer (B.L.) if necessary.

## 3. Results

### 3.1. Residents

All of the youth residents were male, and the majority identified as White (8 out of 9). The average age of the residents was 17.2 (SD = 1.8), with an average of 11.1 years (SD = 1.2) of education. The amount of time spent in the group home ranged from 12–36 months, with an average of 22 months (SD = 7.7).

The thematic analysis identified two overarching themes among the youth residents—*Safety response to COVID-19* and *Socialization changes due to COVID-19*—and three subthemes: *Structure leading to separation*, *Support and belonging amid a pandemic*, and *Competency*.

#### 3.1.1. Overarching Theme 1: Safety Response to COVID-19

The first theme that arose from the resident interviews was safety. The youth residents reflected on the safety precautions put in place because of the pandemic in an attempt to keep COVID-19 out of the group home. Increased safety precautions included handwashing and the use of hand sanitizer, extra cleaning, and restrictions around visitation and leaving the group home campus. Despite the increased safety precautions, many residents shared concerns about contracting the virus while in the group home and their experiences in quarantine. The whole group home went on quarantine because one resident contracted COVID-19 and then it spread. While in quarantine, residents were required to remain in their rooms, separated from staff and others.

“We had to wear masks everywhere we went, and then the staff had to wear a mask around us. So, they were safe from us.”

(P6)

“They got different chemicals to help sanitize then visits would have to be canceled and restrictions.”

(P5)

“Like if you were sick, you would have to quarantine. Even whether or not it was COVID, they made you quarantine away from the rest of the house. Um, I remember I got a cold, and I was in my room for four days because of it. It was irritating.”

(P3)

“But that was one of the safety measures they had put on was they made us separate each other. Like we stayed more distanced, like social distancing.”

(P7)

“They made us wear masks whenever we are indoors, and we have to wear a mask.”

(P8)

“Every morning before school, they take everybody’s temperatures, little temperature going on your forehead, and you have to be less than 97.5 to be able to go in.”

(P3)

Subtheme 1: Structure leading to separation

The group home structure changed drastically because of the increased safety precautions. For example, the youth residents were required to attend school via remote instruction (e.g., Zoom), mental health services changed to remote delivery, daily routines changed significantly, and residents’ free time increased dramatically. For residents, the structure prior to COVID was the most important thing in a group home. From waking up at 6 a.m. for hygiene and breakfast to going to bed at 8:30 p.m., the youth were surrounded by structure. Additionally, school, individual therapy, and group therapy provided a structured environment and encouraged time management. When COVID-19 happened, the structure changed drastically. Since therapists and teachers were not considered “essential” workers, there was an absence in the group home. Once sitting in a class with a live teacher, the students connected to teachers over zoom, which made for a challenge among the staff and youth. Additionally, as a result of more downtime in the group, some youth took the opportunity to be productive and resourceful with their time. Among this population, these kinds of structural changes can be detrimental because of the high-risk situations that accompany boredom and a lack of structure.

“Um, but so I had to like kind of like be a teacher (when school went to Zoom because of COVID).”

(P1)

“It (tele-therapy during COVID) was annoying.”

(P2)

“But most of the time we were stuck in our own room or inside watching TV” (in response to structural changes because of COVID).

(P4)

“Therapy groups changed because of COVID. Um, we would, we would all do it via zoom.”

(P9)

#### 3.1.2. Overarching Theme 2: Socialization Changes Due to COVID

Sex-specific group homes are different from other group homes. The sex-specific group homes included in this study serve a youth population who have committed a sexual crime. Therefore, residents have specific safety measures and limitations due to their crimes and court orders. The residents at these group homes must always be in the line-of-sight of staff, except when using the bathroom. Freedoms for these residents are limited, and isolation is part of the purpose of this type of group home. However, residents can leave the facility with staff and go to the store, the park, or other parts of the community. Residents generally have regular contact with their families based on their progress in the group home.

The residents in these programs tend to enjoy getting out of the group home, always under the supervision of a group home employee, for activities such as shopping, eating, or recreational therapy. Such activities are often integrated into therapeutic efforts, such as providing motivation to make progress, because outings are connected with positive performance in treatment and give the youth an opportunity to maintain a sense of normalcy. Youth look forward to outings, and their loss was expected to be difficult.

Among safety precautions, the lack of visitation (upwards of 6 months) with family members falls under the theme of socialization and safety measures. The interpretation of these themes provided that some participants believed that the ban on visits created a lack of socialization for the family, while other participants acknowledged the safety of having no visits; participants shared that having visits and sending residents on a “pass” or having family members come to the group home perpetuated the risk for contracting COVID-19. Part of the reason for visits is to rebuild trust and the family relationship due to the behaviors that removed the youth from their homes. Given the nature of the population, some of whom are reunifying with their families, the lack of visits and, thus, the lack of socialization did not allow the resident to have a trial placement or practice with family members to assess if the resident was ready to return home. Unfortunately, this restriction created a gap in the development of familial relationships. However, it also created a sense of belonging and a support network between the residents and the staff.

“We really didn’t understand how serious it (COVID) was.”

(P3)

“Outings were emotional outlets but were stopped because of COVID.”

(P1)

“The emotion would be bottled up kind of, and it’s like, I would go home, and I would let out that emotion (pre-COVID). I would talk to my mom. But the emotion would come out when the visit got canceled or the pandemic hit, and we got put on lockdown.”

(P2)

Subtheme 2: Support & belonging amid a pandemic

The residents of these group homes tend to remain in the group home for an average of about two years. They live with other youth and interact with staff regularly. As can be expected, these youth interact more with staff than their family members while in treatment. Additionally, these youth spend nearly every waking hour together—in the home, in school, in group therapy, and sleeping. The youth are placed with similar youth with similar struggles and barriers. Another subtheme identified in this study of youth residing in group homes is support and belonging amid a pandemic. The participants shared the support they felt from staff and others while living in the group home. Group home staff were present to discuss the challenges that youth faced with the restriction of visits, different therapy assignments, and changes in everyday life. Some participants expressed gratitude for the staff presence and even shared that they keep in contact with staff. The increased time that residents spent with each other created a feeling of belongingness. The participants expressed that, with this increased time, they could be their authentic selves without hiding feelings or issues.

“Like I said, I never really looked for support. And now that I have been in the group home, I know what it is like to live life without support, and with support, it is a lot easier to live life. If you have support, I know I was not very close with my mom. In fact, of me my mom really were like butting heads a lot before I got to the group home. But now I feel like we are the closest that I have ever. I am closer now with my mom than I ever have in my whole life. Like the group home really did that. Like change that for me because my mom really has always cared, but I never acknowledged the fact that she cared because I was so set on the fact that I could do it by myself.”

(P3)

“Well, basically we talked about, uh, well, we would like to do in the future with each other, like going out and watching or camping or going hunting or whatever.”

(P2)

“But now that I can see a support asking for help is not a sign of weakness. It is a sign of strength.”

(P3)

“I felt like I got closer with the three boys in my room during those two weeks than I had ever gotten closer with anybody in my entire time at the group home.”

(P3)

“Like when everybody has this good mentality, it is a lot easier to support the people around than when everybody is stressing out about everything all the time. So, like before the pandemic, we were supporting each other. But like we were not supporting each other as much as we were during the pandemic.”

(P4)

“No matter what was going on, it was best to have someone that would listen, whether it was a therapist, a staff, another boy, someone to listen was the best thing, and it was a million times better if they could relate.”

(P2)

“That group setting was really helpful because like they all understand, you did not have to hide things or, you know, it was all out, you know, there was no secret. So helped just you like that was another burden off.”

(P1)

Subtheme 3: Competency

The final subtheme from the youth residents is competency. Because of the change in structure in the group home, some residents were motivated to discharge more quickly while developing therapeutic skills. The researchers believe that competency as a theme developed due to resiliency with the changes forced upon the residents during the pandemic; this created motivation and a drive to complete treatment.

“Oh, I changed how I saw stuff- Um, Kind of hard to explain. It (COVID) just pretty much changed the way and how I saw stuff. Cause like it just gave me different perspectives. Uh, it helped me see like how others feel.”

(P4)

“If I had to do it differently, I would not do it differently, like I would not, it (being in a group home during COVID) was honestly the best thing that happened to me.”

(P1)

“I was doing other stuff because keeping me company from not being able to see my father on the weekends (loss of visits) to just working and doing my treatment to get out of there so I can see my family and playing sports outside and doing all that fun stuff.”

(P6)

“But like that was a crazy point because it (COVID) really showed me, hey, now is the time for me to really utilize the skills that I have learned about emotion, mental management, about self-control, that kind of stuff, empathy, all that stuff that I utilized during quarantine time.”

(P2)

### 3.2. Staff

Given the environment and profession for this particular study, the group home staff tend to be male. This sample included six male staff and two female staff, and half of the staff participants identified as White. The average age of the staff was 37.5 (SD = 14.4), with an average of 13.6 years (SD = 1.5) of education. The amount of time spent working in the group home ranged from 14–168 months, skewing the average to 83.8 months (SD = 70.8).

The thematic analysis identified three overarching themes among the group home staff: *Safety response to COVID-19*, *Increased responsibility*, and *Mental health changes because of a pandemic*.

#### 3.2.1. Overarching Theme 1: Safety Response to COVID-19

All of the group participants shared a safety response to COVID-19 as an overarching theme. In this 24 h surveillance facility, the staff work around the clock. There are morning, swing, and grave shifts, which require communication and accountability for the whereabouts and behaviors of residents. For group home staff, this included enforcing different safety measures and helping residents understand the magnitude of the pandemic. The staff were tasked with implementing safety precautions for residents as well as tending to their own physical health. In this context, the staff were required to implement safety precautions such as handwashing/sanitizing, masks, and social distancing, along with the previously mentioned staffing of residents. Unfortunately, the staff were met with resistance from residents about following safety protocols. These researchers believe that this resistance was due to the lack of understanding regarding the severity of the global pandemic and what the risks were. Despite the resident’s protests, the staff were required to follow mandates in accordance with COVID-19-related policies.

“It was 24/7 just making sure you were safe and safe enough to where the house was safe.”

(P10)

“Monitoring them and making sure nothing, no fights break out, or boundaries being crossed. Like 24-h surveillance.”

(P11)

“So, just monitoring the whole house and dealing with all of that and not wanting to get sick, it was tough.”

(P12)

“When you leave here, you know, you have to be extra careful as well because you don’t want to bring anything back.”

(P17)

“The quarantine part when a lot of kids contracted it. That kind of threw me off a little bit just because I take care of my grandma sometimes outside of here, so I did not want to catch anything and bring it to her.”

(P11)

#### 3.2.2. Overarching Theme 2: Increased Responsibility

A great deal of responsibility was placed on the group home staff. Job duties changed drastically as the pandemic unfolded; for example, staff members took on more shifts to cover for others, staff members were tasked with setting up therapy zoom visits, zoom family visits, and zoom sessions for school. Essentially, the staff became the backbone of the group home and took on many different roles such as surrogate teachers and therapists while also maintaining their job duties. As clinicians in the group home (W.H. & A.A.), we were not seen as “essential” workers who needed to be there in-person. We (W.H. & A.A.) heard from residents, staff, and guardians about the various struggles in the group home because of COVID-19, and we occasionally witnessed the increased mental health needs of residents and staff because of the pandemic. Additionally, the staff took on the responsibility of supporting residents with elevated mental health concerns and elevated behaviors such as outbursts. Regardless of their fears, the staff expressed an obligation and commitment to show up to work. Some staff members shared fear about bringing COVID-19 to work, demonstrating a sense of responsibility to the organization and proper health precautions.

“If I did not feel safe enough like if I wanted to take time off, I could.”

(P12)

“The boys did not understand quarantining. They were like, this is stupid. We do not need this.”

(P14)

“I did not take time off because I felt like the kids needed somebody here.”

(P11)

“Trying to get through to them that not only is it dangerous for them to be outside the house, but it is dangerous for their families too.”

(P14)

“That is why through the pandemic and stuff like that, I was always making sure that we could make something work.”

(P10)

#### 3.2.3. Overarching Theme 3: Mental Health Changes Because of a Pandemic

Along with the rest of the world, mental health became a challenge for the group home staff. Because the staff were considered the organization’s backbone, there were challenges regarding mental health in the context of taking care of their mental health. Stress was at an all-time high, along with fears and exhaustion. Some staff described their experiences as burnout. Self-care was identified as a way to tend to mental health among the staff.

“Quarantine was nerve-wracking.”

(P11)

“I got burnt out” [working during COVID-19].

(P16)

“I can speak for most staff when I say like there are days when it is taxing on my mental health, hearing stories or just everyday behaviors that these guys have.”

(P13)

“It was just kind of nice to get a, you know, like a five-minute breather from the kids sometimes.” [In reference to working during COVID-19.]

(P14)

“I am a high risk” [to contract COVID-19].

(P12)

### 3.3. Guardians

Most of the guardians of the youth residents were female (10 of 13), including mothers and grandmother caregivers. All of the guardian participants identified as White. The average age of the guardians was 45.1 (SD = 5.4), with an average of 13.9 years (SD = 2.1) of education. The amount of time spent having a youth in the group home ranged from 12–60 months, skewing the average to 24.2 months (SD = 13.4).

The thematic analysis identified three overarching themes among the guardians of the youth residents: *Safety response to COVID-19*, *Belief in a mental health impact on the child*, and *Communication during a pandemic*.

#### 3.3.1. Overarching Theme 1: Safety Response to COVID-19

Like the residents, the response to safety arose as an overarching theme for the guardians. The parents spent time sharing their perspectives on how the safety concerns during COVID-19 changed for their youth. Although the parents were not living in the group home, they were provided with up-to-date information about how safety was being managed.

“At that point, he had one roommate, and the staff would bring their food, set it at the door, and then step back, so they were maintaining distance.”

(P23)

“As long as the state, as long as the governor was saying limit, limit gatherings, they kept a locked down. Um, so it was a good six months.”

(P26)

“They were all wearing masks, um, when they were around people. They did not take them to the stores.”

(P23)

“But then when I was like hearing of, um, staff members getting COVID and bringing it into the home and the like all the boys got sick, I kind of lost it.”

(P22)

“So, because of COVID, UM, we were just doing phone calls.”

(P30)

“There is a sense of relief and feelings of more safety.”

(P24)

#### 3.3.2. Overarching Theme 2: Belief in a Mental Health Impact on the Child

As researchers coded the transcripts, the projection of parents’ mental health on children during the pandemic was brought to light. According to the parents, various factors were found to have impacted the residents’ mental health. Though the parents expressed the mental health impact on the residents, the researchers also acknowledge the mental health challenges among the parents and grandparents. The parents wanted to have visits with their child and were unable to do so, which could have led to the belief that the residents’ mental health was being impacted negatively. Because of the lack of knowledge regarding private conversations between the parents and residents, the degree of the mental health impact on their children is unclear; all that the researchers are aware of is the information provided through semi-structured interviews. Additionally, this is supported by the fact that mental health was not a theme among the youth residents. The residents did not share that their mental health was impacted any more than it was before the pandemic.

“He never met with his therapist face to face ever the whole time he was at the group home. Which was not the therapist’s fault and not, you know what I mean? But I think it really negatively impacted his ability to like engage with that therapist. And so, it was less personal than I think it could have been. And I think it could have had a bigger impact than it did because of remote therapy sessions.”

(P27)

“I know it was a huge difference in his mental stability to be able to have that interaction.” [In reference from going to therapist presence every day to absence by therapists.]

(P23)

“Oh yea, he would definitely would have more of a, he would have his days when he just really was solid.” [In reference to mental health being stable]

(P19)

“I could definitely tell that it made him sad and that he was struggling.”

(P28)

“Everything else had had to be very trying, especially for people who already deal with depression and stuff like that.”

(P19)

#### 3.3.3. Overarching Theme 3: Communication during a Pandemic

For a group home to succeed, there needs to be a great deal of communication with many other stakeholders such as caseworkers, juvenile probation officers, and parents. Updates to these groups are often made weekly through progress reports and family therapy sessions. The guardian participants in our study reported notable challenges with the communication between them and the organization. The parents felt like they were not communicated with about the decisions being made, the reasons for the decisions, and updates on their child. They felt frustrated, helpless, and uninformed. The other facet of the communication theme included increased communication between the parents and children. The group home allowed residents to call every day, whereas before, they called three times a week. The group home also allowed for family visits via Zoom, which led to increased contact between families, thus increasing healthy familial relationships. Although the consensus was that communication increased, some guardians shared that their child struggled to keep in contact.

“There really wasn’t communication.”

(P19)

“So, our communication went down a lot when the pandemic hit.”

(P21)

“We could not see our family, though we called our family every day so we could talk to them.”

(P3)

“With the pandemic, it was only phone calls, and it was like pulling teeth out sometimes to get him to call.”

(P30)

“I was late to communication for a little while as a pandemic was only phone calls.”

(P22)

“Um, we did more family sessions, and sometimes that was so much communication.”

(P21)

## 4. Limitations

This study is not without limitations. First, our sampling method was a convenience sample including any stakeholder involved in the group home if they met the inclusion criteria. A second limitation of the current study is that group homes for youth who have sexually assaulted other youth are very specific, limiting generalizability. Further, our study was rather homogeneous, which also limits generalizability.

A key strength of our study is that it yielded some insight into the lived experiences of several group home stakeholders during the pandemic. We interviewed residents, gaining an in-depth perspective on life in a group home during a pandemic. Additionally, we interviewed staff members of the group home, as their role is vastly different. Finally, we interviewed guardians who had a child in a group home and gained access to an unparalleled and unique perspective. We achieved a fuller picture from seeking insights from multiple participants rather than just one role. Another strength is our overall sample size (N = 30). Finally, this study was conducted with participants who experienced the group home both before and during the pandemic.

## 5. Conclusions

This study provided subjective evidence of changes experienced by youth residents, staff, and guardians of youth in a group home during the COVID-19 pandemic. Youth residents, staff, and guardians experienced various daily changes and were forced to adapt to new living situations in the group home due to the pandemic. The participants of this study shared their experiences and provided a glimpse of what it is like living in, working in, and having a child in a group home during a global pandemic. While some participants described the pandemic as perpetuating isolation and aloneness, others addressed the positive shift they experienced in terms of their therapy and mental health. Our findings can inform ways of responding to the needs of residents, staff, and guardians during times when normal operating procedures are significantly disrupted.

An Assistant Professor of Psychology at Widener University defines resilience as “a sense of hardiness” [12]. Going further, she shares that it is a person’s confidence that they can rely on their internal resources to overcome challenges [12]. In this study, individuals described their challenges and the changes they endured, influencing them to use resilience to overcome these challenges. It was observed that what these participants were experiencing was resilience. Our findings demonstrate human resilience on multiple levels. The participants demonstrated personal growth during this time. For the parents, they were already away from their children given the nature of the group home; this, on top of the stress of a pandemic, allowed the parents to build trust with other individuals who were supervising their youth. The staff were on the front lines of being exposed to COVID-19; enforcing safety measures while dealing with their own mental and physical health created flexibility and strength. The residents were in the middle of the pandemic in the group home, relying on others to keep them safe and using their own internal resources to keep themselves safe and healthy.

Furthermore, this study provides insight on the basis of which group homes/organizations can plan and prepare for crises of a great magnitude in order to maintain mental health, socialization, and skills for youth in these settings. Additionally, the findings inform the need to create more effective communication strategies among all the parties involved. These youth and guardians are already isolated and stigmatized due to their history and their child’s offenses, respectively. Finally, though this article contributes to the current literature, further in-depth research is needed to explore the impact on youth, staff, and guardians in the context of a group home during COVID-19 and the effects it may have.

## References

[1] American Academy of Pediatrics (2022). Children and COVID-19: State-Level Data Report. https://www.aap.org/en/pages/2019-novel-coronavirus-covid-19-infections/children-and-covid-19-state-level-data-report/.

[2] Braun V., Clarke V., Cooper H., Camic P.M., Long D.L., Panter A.T., Rindskopf D., Sher K.J. (2012). Thematic analysis. APA Handbook of Research Methods in Psychology.

[3] Costa M., Matos P.M., Santos B., Carvalho H., Ferreira T., Mota C.P. (2021). We stick together! COVID-19 and psychological adjustment in youth residential care. Child Abus. Negl..

[4] Font S. (2020). The impact of the COVID-19 pandemic on children in foster care: Exploring the issues and potential solutions. https://covid19.ssri.psu.edu/articles/impact-covid-19-pandemic-children-foster-care.

[5] Haffejee S., Thembekile-Levine D. (2020). ‘When will I be free’: Lessons from COVID-19 for child protection in South Africa. Child Abus. Negl..

[6] Hodgdon H.B., Blaustein M., Kinniburgh K., Peterson M.L., Spinazzola J. (2016). Application of the ARC Model with Adopted Children: Supporting Resiliency and Family Well Being. J. Child Adolesc. Trauma.

[7] Kinniburgh K., Blaustein M., Spinazzola J., Van der Kolk B.A. (2005). Attachment, Self-Regulation, and Competency. Psychiatr. Ann..

[8] Montserrat C., Garcia-Molsosa M., Llosada-Gistau J., Sitjes-Figueras R. (2021). The views of children in residential care on the COVID-19 lockdown: Implications for and their well-being and psychosocial intervention. Child Abus. Negl..

[9] Parry S., Williams T., Oldfield J. (2022). Reflections from the forgotten frontline: ‘The reality for children and staff in residential care’ during COVID-19. Health Soc. Care Community.

[10] Rosenberg R., Sun S., Flannigan A., O’Meara M. (2022). Impact of COVID-19 among young people currently and formerly in foster care. Child Abus. Negl..

[11] Wilke N.G., Howard A.H., Goldman P. (2020). Rapid return of children in residential care to family as a result of COVID-19: Scope, challenges, and recommendations. Child Abus. Negl..

[12] Smith E.E. (2021). Pandemic brought out something positive for some people- resilience. https://www.washingtonpost.com/health/pandemic-resilience/2021/06/18/a82d69fc-a9f0-11eb-8d25-7b30e74923ea_story.html.

